# Analysis of the function of IL-10 in chickens using specific neutralising antibodies and a sensitive capture ELISA

**DOI:** 10.1016/j.dci.2016.04.016

**Published:** 2016-10

**Authors:** Zhiguang Wu, Tuanjun Hu, Lisa Rothwell, Lonneke Vervelde, Pete Kaiser, Kay Boulton, Matthew J. Nolan, Fiona M. Tomley, Damer P. Blake, David A. Hume

**Affiliations:** a The Roslin Institute and Royal (Dick) School of Veterinary Studies, University of Edinburgh, Easter Bush, Midlothian EH25 9RG, UK; b Department of Pathology and Pathogen Biology, Royal Veterinary College, University of London, Hatfield AL9 7TA, UK

**Keywords:** Chicken, Interleukin-10, Monoclonal antibody, Capture ELISA, Neutralising antibody

## Abstract

In mammals, the inducible cytokine interleukin 10 is a feedback negative regulator of inflammation. To determine the extent to which this function is conserved in birds, recombinant chicken IL-10 was expressed as a secreted human Ig Fc fusion protein (chIL-10-Fc) and used to immunise mice. Five monoclonal antibodies (mAb) which specifically recognise chicken IL-10 were generated and characterised. Two capture ELISA assays were developed which detected native chIL-10 secreted from chicken bone marrow-derived macrophages (chBMMs) stimulated with lipopolysaccharide (LPS). Three of the mAbs detected intracellular IL-10. This was detected in only a subset of the same LPS-stimulated chBMMs. The ELISA assay also detected massive increases in circulating IL-10 in chickens challenged with the coccidial parasite, *Eimeria tenella*. The same mAbs neutralised the bioactivity of recombinant chIL-10. The role of IL-10 in feedback control was tested *in vitro*. The neutralising antibodies prevented IL-10-induced inhibition of IFN-γ synthesis by mitogen-activated lymphocytes and increased nitric oxide production in LPS-stimulated chBMMs. The results confirm that IL-10 is an inducible feedback regulator of immune response in chickens, and could be the target for improved vaccine efficacy or breeding strategies.

## Highlights

• Five mouse mAbs to chicken IL-10 was produced.
• Two capture ELISA assays were developed to quantify native IL-10.
• Three mAbs (ROS-AV162, 163 and 164) neutralised the bioactivities of chIL-10.
• Anti-IL-10 antibodies detected intracellular IL-10 in only a subset of LPS-stimulated macrophages.
• *Eimeria* infection of chickens produced a massive increase in circulating IL-10.

## 1 Introduction

Interleukin-10 (IL-10) is an anti-inflammatory cytokine that controls the nature and extent of inflammatory responses during infection with viruses, bacteria, fungi, protozoa and helminths (Couper et al., 2008, Moore et al., 2001) and has a particular central role in intestinal immunity and homeostasis (Manzanillo et al., 2015). IL-10 was first described as an inhibitor of cytokine synthesis in mice, a product of the TH2 subset of T cells that inhibited synthesis of proinflammatory cytokines by TH1 cells (Fiorentino et al., 1989). IL-10 is now recognised as a multifunctional cytokine produced by many immune cell types including macrophages, monocytes, dendritic cells (DCs), TH1, TH2, TH17 and regulatory T cell subsets and B cells and a feedback regulator of diverse immune responses to infections (Reviewed in Couper et al., 2008, Moore et al., 2001, Saraiva and O'Garra, 2010). Accordingly, vaccine efficacy in mammals has been associated with genetic variation in IL-10 production, and anti-IL-10 treatment has been shown to increase the nature, magnitude and efficacy of vaccine responses against a diversity of pathogens (Chen et al., 2014, Darrah et al., 2010, Pitt et al., 2012, Roberts et al., 2005, Stober et al., 2005).

Infectious diseases are a major threat to intensive poultry production. There is less evidence of the function of IL-10 in birds. The cDNA of chicken IL-10 (chIL-10) was isolated from cecal tonsils of *Eimeria tenella*-infected chickens (Rothwell et al., 2004) and the expressed protein product inhibited IFN-γ synthesis by mitogen-activated lymphocytes (Rothwell et al., 2004). Inducible expression of IL-10 mRNA has been described in viral, bacterial and parasitic infections of birds (Barjesteh et al., 2013, Humphrey et al., 2014, Parvizi et al., 2015). However, the lack of reagents has prevented analysis of the production and function of IL-10 protein. In the current study we describe the generation of monoclonal antibodies to chIL-10 and their applications in western blot, capture ELISA and neutralising assays. The results indicate that IL-10 has similar functions in birds as in mammals. As predicted from the original studies on infected birds (Rothwell et al., 2004), *Eimeria* infection of chickens produced a massive increase in circulating IL-10, providing a biomarker for disease status. Accordingly, we suggest that manipulation of IL-10, either through genetic or therapeutic intervention, could have application in reducing disease burden in chickens.

## 2 Materials and methods

### 2.1 Expression and purification of recombinant chIL-10-Fc and recombinant chIL-10-V5H6 protein

ChIL-10 cDNA was sub-cloned into the vector, pKW06 (John Young, unpublished) to express the protein in mammalian cells with a C-terminal human IgG1 Fc tag. For screening purposes, to eliminate anti-human Ig antibodies, a V5His tagged chIL-10 was produced in the vector pKW08 (John Young and Tuanjun Hu, unpublished). ChIL-10 cDNAs (Rothwell et al., 2004) were expressed with the native signal peptide allowing secretion of the fusion proteins. Both constructs were expressed in COS-7 cells following transfection using the DEAE-dextran method (Rothwell et al., 2004). Recombinant chIL-10-Fc protein was purified using a HiTrap Protein G affinity column and the chIL-10-V5H6 protein with a HisTrap excel column (GE Healthcare Life Sciences).

### 2.2 Monoclonal antibody production, isotyping, purification, and labelling

Immunisation with chIL-10-Fc and fusion to generate hybridomas was carried out by Dundee Cell Products (DCP, Dundee, UK). Following fusion, hybridoma cultures were tested with recombinant chIL-10-V5H6 by dot-blot. Supernatants from these cultures were screened by ELISA with chIL-10-V5H6 and chIL-10-Fc and Western-Blot. Positive cultures were selected for further cloning. The antibody isotype was determined using the IsoStrip Mouse Monoclonal Antibody Isotyping Kit (Roche). Monoclonal hybridomas were cultured in D-MEM with 10% Ig-depleted FBS. Monoclonal antibodies were purified using HiTrap Protein G affinity columns and dialysed against PBS using 30 kDa molecular weight cut-off (MWCO) Slide-A-Lyser cassettes (Pierce, ThermoFisher Scientific). The concentrations of mAbs were determined by absorbance at 280 nm with a Nanodrop and then aliquots of the purified antibodies were biotinylated using Sulfo-NHS-LC-LC-biotin (Thermo Scientific). All procedures were performed according to the manufacturer's instructions.

### 2.3 Western blot

Recombinant chIL-10-V5H6 was treated with SDS-PAGE reducing buffer, denatured for 5 min at 100 °C and loaded onto a 4–15% pre-cast Mini-PROTEAN TGX Gels (Bio-Rad) and transferred onto a nitrocellulose membrane (Immunobilon-P, Millipore) using a Trans-Blot Semi-Dry Electrophoretic Transfer Cell (Bio-Rad). After blocking with 0.5% skimmed milk power/PBS solution, the membrane was stained with 1.0 μg/ml of each mAb, followed by incubation with goat anti-mouse IgG1-horseradish peroxidase (Southern Biotec) diluted in 0.5% skimmed milk powder/PBS. Detection was carried out using enhanced chemiluminescence (ECL) (GE Healthcare Life Sciences), according to the manufacturer's instructions.

### 2.4 Detection of chicken IL-10 by indirect ELISA and development of capture ELISA assays

Indirect ELISA was performed as described previously (Rothwell et al., 2001). Briefly, assay plates (Nunc Immuno MaxiSorp, Thermo Electron LED) were coated with recombinant chIL-10 or control protein in Carbonate/Bicarbonate buffer and incubated overnight at 4 °C. Plates were washed in PBS containing 0.05% Tween-20 (PBS-T) and blocked with 0.5% casein/PBS at room temperature (RT) for 1 h. Purified mAb was added to the plate at a 1.0 μg/ml and incubated at RT for 1 h. After three washes with PBS-T, plates were incubated with goat anti-mouse IgG-HRP at RT for 1 h. After a further three washes, plates were visualised by TMB substrate (Thermo Scientific) and reaction was stopped by 2 N H_2_SO4. Plates were read at 450 nm in a SpectraMax 250 microplate spectrophotometer system (Molecular Devices, Sunnyvale, CA, USA). Two capture ELISA assays was developed using ROS-AV162 or 164 as capture antibody and biotinylated ROS-AV163 as detecting antibody. Briefly, assay plates were coated with capture antibody ROS-AV162 or ROS-AV164 at 4 μg/ml overnight at 4 °C. Plates were washed and blocked as in the indirect ELISA. Plates were incubated with recombinant IL-10 standards, or test samples at RT for 1 h, then washed and incubated with biotinylated detecting antibody ROS-AV163 at 1 μg/ml at RT for 1 h. After three washes, plates were incubated with streptavidin–HRP (1:10,000, Pierce) for a further hour at RT before adding substrate 1-step TMB (Thermo Scientific) and then sulfuric acid stop solution. Absorbance was read at 450 nm.

### 2.5 Detection of IL-10 production by bone marrow derived macrophages (BMMs)

Chicken BMMs were cultured from bone marrow cells from ED20 Novogen embryos in the presence of recombinant chicken CSF-1 as previously described (Garceau et al., 2010). On day 7 of culture, cells were stimulated with 0.5 μg/ml of LPS (*Eimeria coli* serotype 055:B5, Sigma) to induce IL-10 expression. Cell culture supernatant from BMMs cells was collected at various times and IL-10 was detected using the capture ELISA. To detect intracellular IL-10 protein, the BMM cells were stimulated with 0.5 μg/ml of LPS for 2 h before Brefeldin A (10 μg/ml, Sigma-Aldrich) was added to the culture to block secretion. Cells were resuspended in Fixation/Permeabilization solution (BD Bioscience) for 20 min at 4 °C, washed twice with 1× BD Perm/Wash™ buffer, and incubated with ROS-AV160-164 at 1.0 μg/ml in 1× BD Perm/Wash™ for 30 min at 4 °C. Cells were washed as before and resuspended in 0.4 μg/ml Alexa Fluor 647 goat anti-mouse IgG1 (Invitrogen/A-21240). Cells were washed then three times before analysing on FACScalibur (BD Bioscience).

### 2.6 Infection of chicken with *E. tenella* and measurement of circulating IL-10

Commercial broiler birds (Cobb 500) were reared under SPF conditions from day of hatch and screened by faecal flotation to confirm the absence of prior Eimeria infection. Birds were inoculated with a low (4000) or high (35,000) dose of *E. tenella* Houghton strain sporulated oocysts, or mock dosed using sterile phosphate buffered saline, at 4 weeks post hatch (n = 6). Serum was taken following brachial vein blood collection 2 days prior to infection (−2 dpi), 5 days post infection (dpi) and 8 dpi. This study was carried out in strict accordance with the Animals (Scientific Procedures) Act 1986, an Act of Parliament of the United Kingdom, following approval by the Royal Veterinary College Ethical Review Committee and the United Kingdom Government Home Office under the project licence 70/7781. The levels of IL-10 in the sera were measured by capture ELISA using ROS-AV164 as capturing antibody and biotinylated ROS-AV163 as detecting antibody.

### 2.7 IL-10 bioassay and mAb neutralisation assay

ChIL-10 bioassay was performed as described previously (Rothwell et al., 2004). Briefly, splenocytes were isolated from 8 week old birds over Histopaque and resuspended at 5 × 10^6^ cell/ml in DMEM containing 2 mg/ml BSA, 1% l-glutamine, 1 U/ml penicillin, and 1 μg/ml streptomycin. Cells were added to round-bottom 96-well plates (100 μl/well) containing serial 5-fold dilutions of rchIL-10 or negative controls (including control protein tagged with HuIgG1 Fc, control protein tagged withV5H6, pCIneo supernatant), in a final volume of 200 μl in the presence of 0.5 μg/ml of ConA (Sigma-Aldrich) or no ConA. Plates were incubated at 41 °C for 72 h for analysis of IFN-γ content in the supernatant by capture ELISA (IFN-gamma chicken antibody pair, Novex, Life technologies, UK) as per manufacturer's instructions. For neutralisation assay, rchIL-10 was pre-incubated with 2-fold diluted mAbs or a control mAb with a starting concentration of 4 μg/ml at 37 °C for 2 h before adding lymphocytes and ConA.

### 2.8 Nitric oxide assay

Chicken BMMs were cultured on 96 well plates as above. On day 7 of culture, cells were stimulated with 0.5 μg/ml of LPS (*E. coli* serotype 055:B5, Sigma) with or without mAbs ROS-AV160 to 164. Supernatant was collected at 24 h after treatment to measure nitrite, a stable metabolite of nitric oxide, by Griess assay. Briefly, 100 μl of culture supernatant from each well was transferred to a new flat-bottom 96-well plate. Griess reagent was prepared by mixing equal volumes of components A (0.2% α-napthyl ethylene diamine dihydrochloride in dH_2_O) and B (2% sulfanilimide in 5% phosphoric acid in dH_2_O). Equal amount of Griess reagent was added to each wells. After 10 min incubation at room temperature, the plates were read at 570 nm in a SpectraMax 250 microplate spectrophotometer system (Molecular Devices, Sunnyvale, CA, USA). Sodium nitrite (Promega) was used as a standard to determine nitrite concentrations.

## 3 Results and discussion

### 3.1 Production of mouse anti-chicken IL-10 monoclonal antibodies

**Fig. 1:**
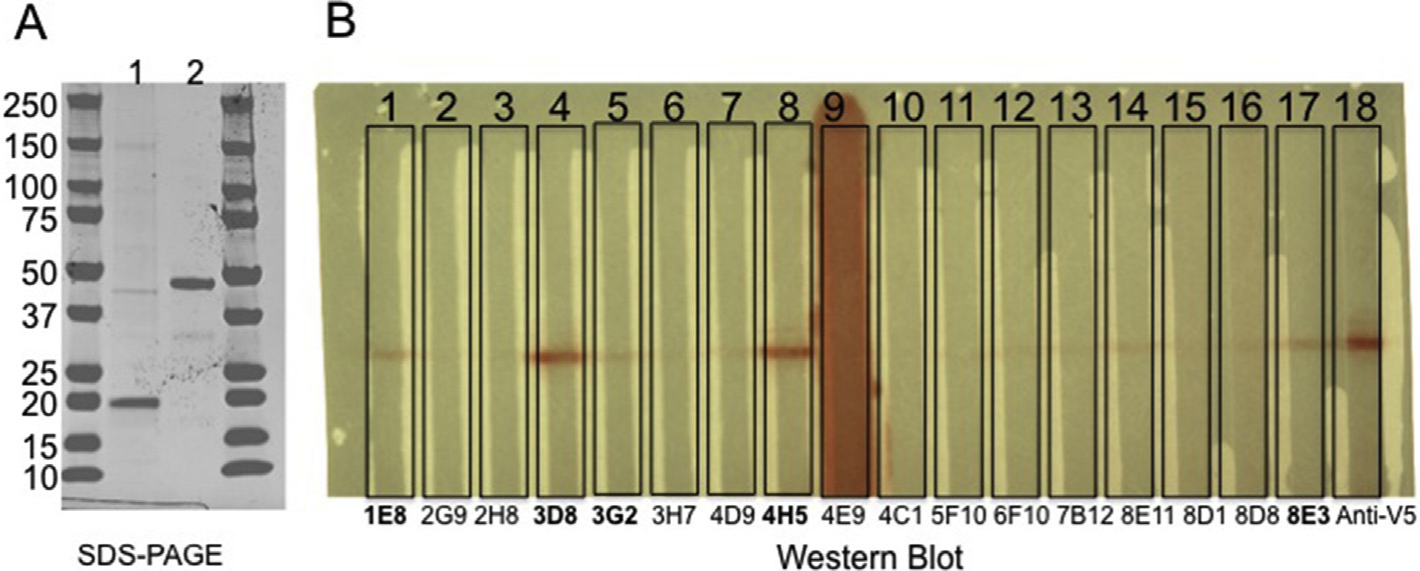
(A) SDS-PAGE showing the purity and molecular weight of chIL-10-V5H6 (lane 1) and chIL-10-Fc (lane 2). (B) Screening of hybridoma supernatant by WB. Clone 1E8, 3D8, 4H5, 8E11 and 8E3 recognised chIL-10-V5H6 by WB. These five clones were selected to produce monoclonal antibodies.

**Fig. 2:**
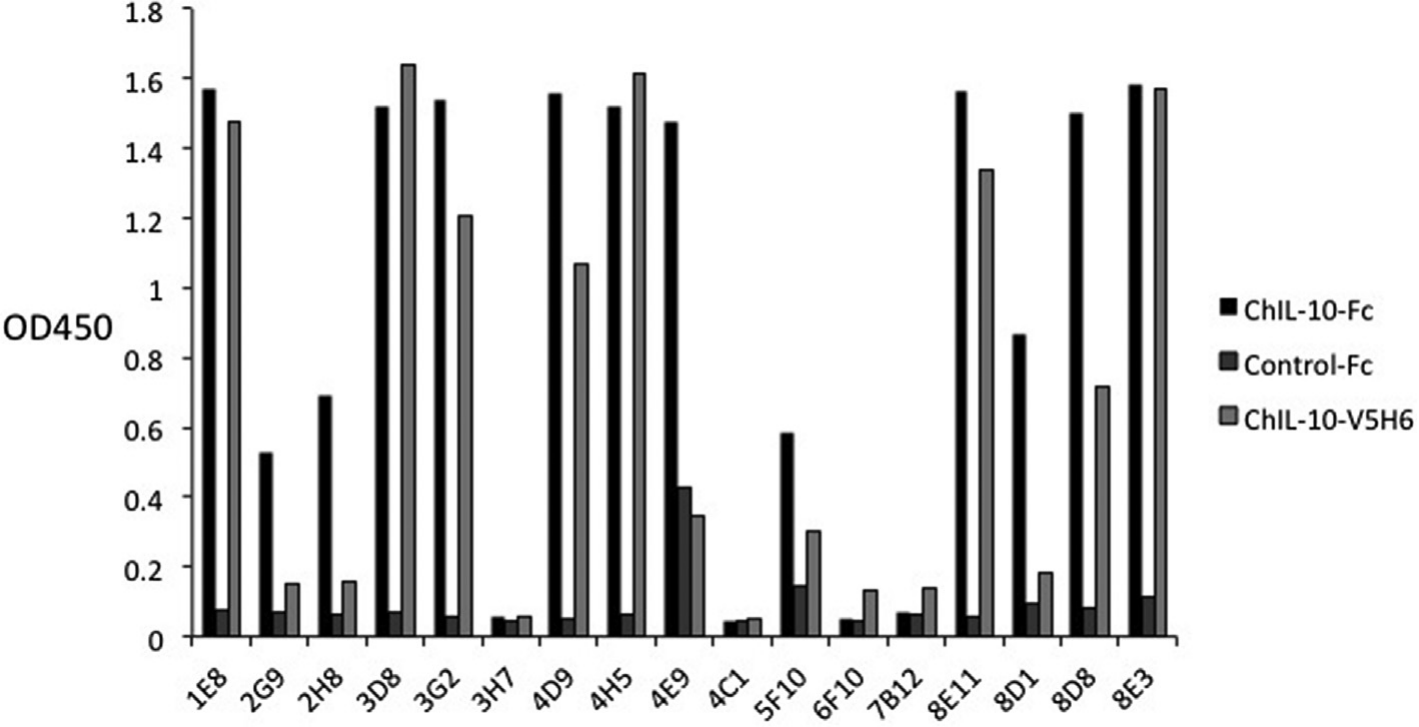
Screening of hybridoma supernatant by ELISA. Values represent the mean of duplicate wells. Clone 1E8, 3D8, 3G2, 4D9, 4H5, 8E11, 8D8 and 8E3 recognised chIL-10 but not control-Fc.

The predicted molecular weight of the expressed chIL-10-Fc and chIL-10-V5H6 proteins are 46.53 kDa and 20.97 kDa. Following expression and purification as described in the Methods, the purity and molecular weight were confirmed by SDS-PAGE (Fig. 1A). The purified chIL-10-Fc fusion protein was used to immunise mice for generation of mAbs. Seventeen initial hybridoma supernatants reactive with chIL-10-Fc were tested for specific binding to chIL-10 by WB and ELISA (Figs. 1B and 2). Eight supernatants recognised chIL-10 but not control-Ig by ELISA and five of these also bound chIL-10-V5H6 by WB. The corresponding lines were cloned and five IgG1 monoclonal antibodies (ROS-AV160 (4H5), ROS-AV161 (3D8), ROS-AV162 (8E11), ROS-AV163 (8E3), ROS-AV164 (1E8)) were produced.

### 3.2 Characterisation of mAbs by indirect ELISA and western blot (WB)

**Fig. S1:**
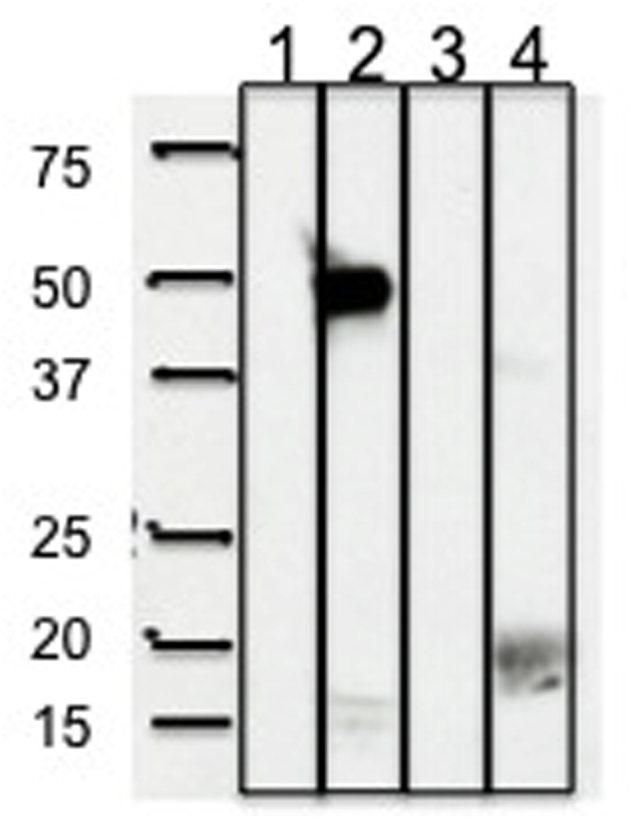
Specificity of mouse anti-chIL-10 mAb as demonstrated by WB against rchIL-10-Fc, control-Fc, chIL-10-V5H6 and control-V5H6. All five monoclonal antibodies specifically recognised recombinant chIL-10 via WB. Result using ROS-AV163 was shown here. Secondary antibody was goat anti-mouse IgG-HRP and detection was by ECL. Lane 1, control-Fc; Lane 2, Purified chIL-10-Fc; lane 3, control-V5H6; Lane 4, purified chIL-10-V5H6.

**Fig. 3:**
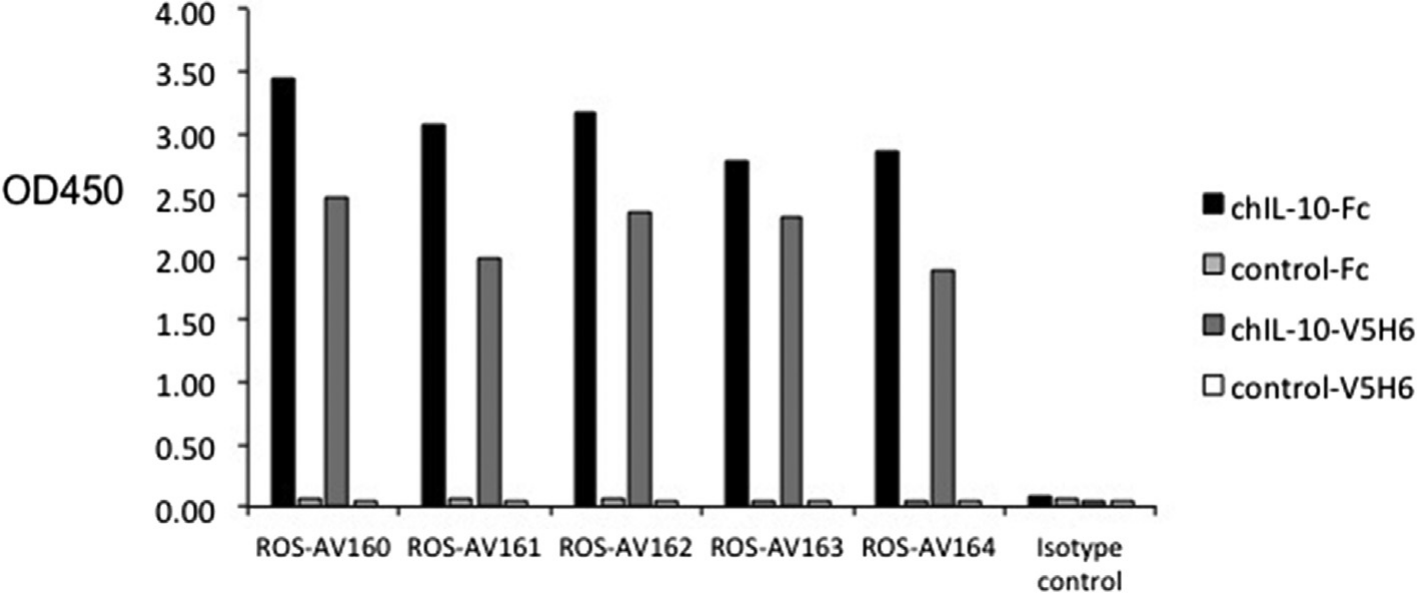
Specificity of mouse anti-chIL-10 mAb as demonstrated by indirect ELISA against rchIL-10-Fc, control-Fc, chIL-10-V5H6 and control-V5H6. All five monoclonal antibodies specifically recognised recombinant chIL-10 not Fc or V5H6 tagged control protein. Values represent the mean of duplicate wells.

All five monoclonal antibodies reacted specifically with recombinant chIL-10, by ELISA (Fig. 3) and bound both the 47 kDa of chIL-10-Fc and 21 kDa of chIL-10-V5H6 (Supplemental material Fig. S1). Based upon epitope blocking experiments (data not shown), ROS-AV160 and ROS-AV161 recognise the same or closely-related epitopes on IL-10, whereas ROS-AV162, ROS-AV163 and ROS-AV164 recognised different epitopes. Accordingly, these antibodies in combination were considered appropriate to establish a capture ELISA.

### 3.3 Detection of native chIL-10 by capture ELISA and intracellular staining

**Fig. 4:**
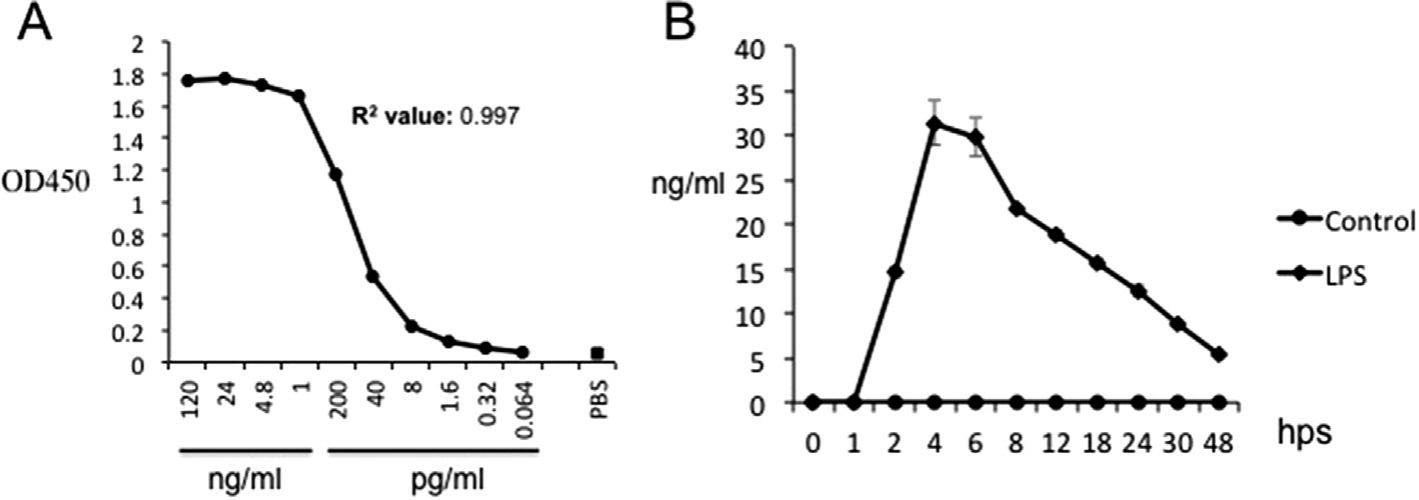
(A) Capture ELISA with ROS-AV164 as the capture antibody at 4 μg/ml and biotinylated ROS-AV163 at 1 μg/ml as detecting antibody. Recombinant chIL-10-Fc was used as standard. PBS was used as negative control. Values represent the mean of duplicate wells. (B) Native chIL-10 in LPS-stimulated BMMs culture in a time course was detected by the above capture ELISA. Values represent the mean of three independent experiments. Error bars represent standard error of the mean.

Two capture ELISA assays for chIL-10 were developed using different combinations of mAbs. Both assays give the same sensitivity so only data from one assay (capturing with ROS-AV164 and detecting with biotinylated ROS-AV163) was carried forward. The sensitivity range of this assay was between 8 pg/ml to 1 ng/ml (Fig. 4A). Many cells of the innate immune system, including macrophages, dendritic cells, mast cells, NK cells, eosinophils and neutrophils express IL-10 (Saraiva and O'Garra, 2010). In mammals at least, macrophages can express IL-10 *in vitro* following activation of specific PRRs with LPS (Chang et al., 2007) or other TLR agonists (Boonstra et al., 2006). In the chicken, activation of TLR3 and TLR21 through poly I:C (double-stranded RNA) or CpG-ODN (a CpG-motif containing oligodeoxydinucleotide) respectively induced IL-10 mRNA in monocytes (He et al., 2012). In splenocytes, IL-10 mRNA was induced transiently in response to various bacterial lipopolysacharides (Barjesteh et al., 2013).

**Fig. 5:**
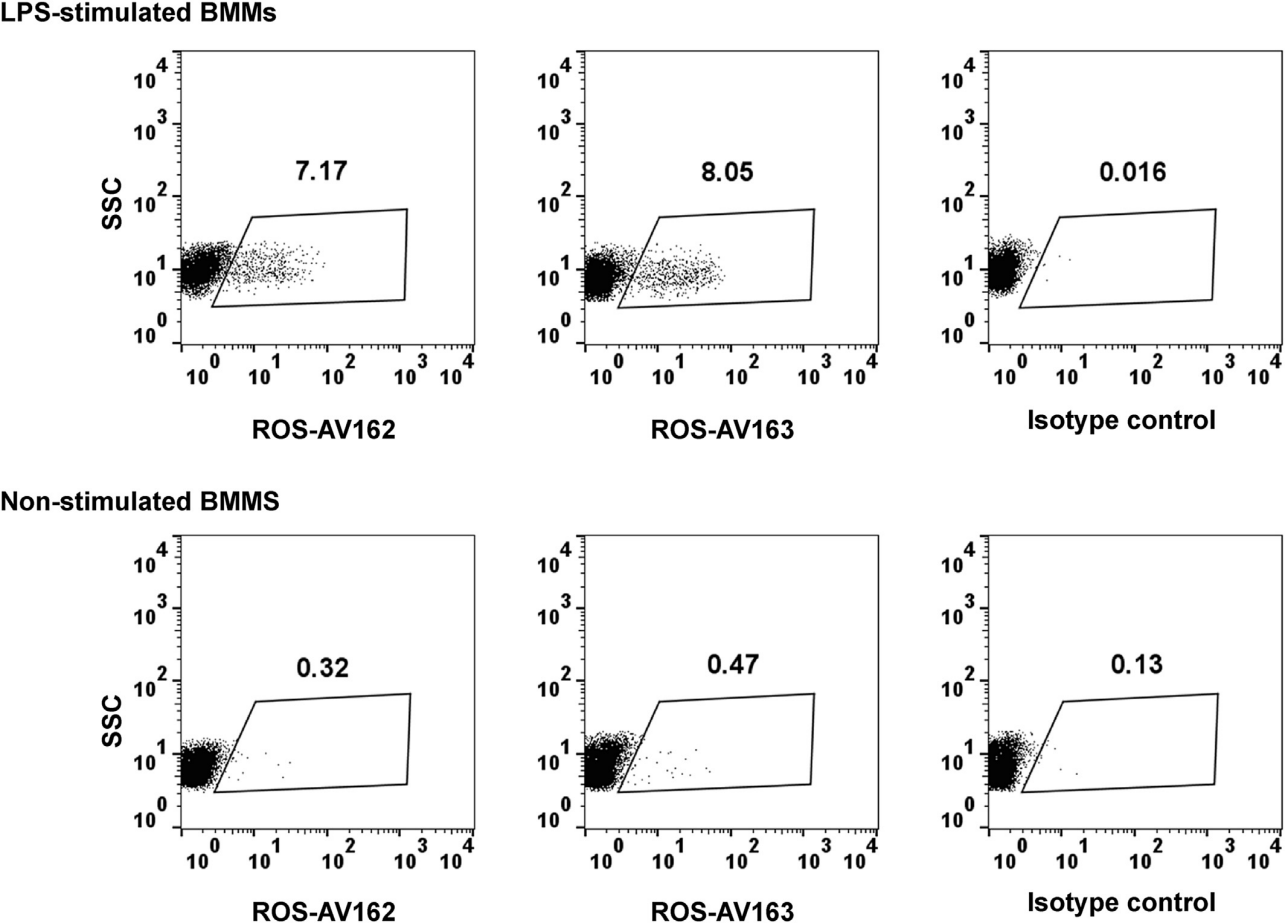
Detection of intracellular chIL-10 in LPS-stimulated chBMMs. After 7-days culture, ChBMMs were stimulated with LPS for 2 h or non-stimulated before adding Brefeldin A for further 4 h. Cells were fixed, permeabilised and stained as described in materials and methods. Cells debris were gated out. SSC: side scatter.

The ELISA was used to detect secretion of chIL-10 by LPS-stimulated BMMs (Fig. 4B). ChIL-10 in the supernatant was rapidly induced from 2 h post stimulation with LPS, reached a maximum after 4 h and went down gradually. In stimulated human (Xue et al., 2015) and mouse macrophages (Ravasi et al., 2002), only a subset of cells express each individual inducible cytokine. Intracellular cytokine detection by flow cytometry allows studying cytokine production at single cell level together with other cytokines or cell surface markers. The anti-chIL-10 antibodies were used to detect intracellular chIL-10. ChBMMs were stimulated with LPS for 2 h and BFA was used to block the secretion of chIL-10 (Fig. 5). Both ROS-AV162 and 163 detected intracellular chIL-10, but surprisingly only around 10% of the LPS-stimulated chBMMS were positive. This result suggest that the secreted IL-10 may be produced by only a subset of the stimulated macrophages, and might therefore act in a paracrine manner on neighbouring cells.

### 3.4 Detection of circulating IL-10 after *E. tenella* infection

**Fig. 6:**
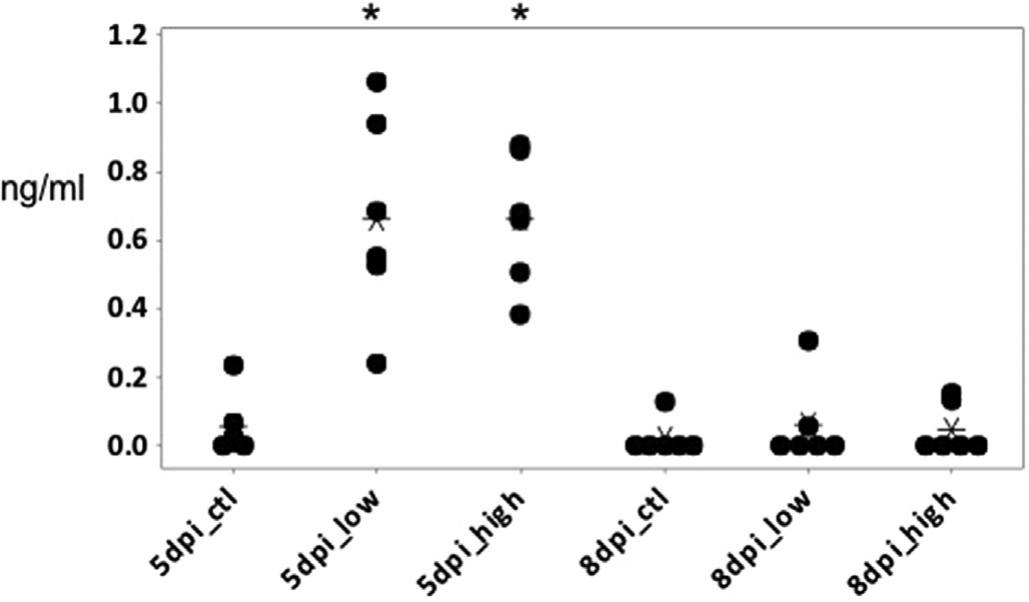
Circulating IL-10 was detected in the serum from chicken infected with *E. tenella* at 5 dpi. Six birds were used for each group. Student's t-test was used to determine significance (P < 0.05, indicated by an asterisk) of IL-10 production in infected birds, when compared to the relevant control.

IL-10 is a key immunoregulator during infection with pathogens including intracellular protozoa (Gazzinelli et al., 1996, Wilson et al., 2005). As in mammalian species, chicken IL-10 has emerged to be a crucial player in the Th bias during infection with *Eimeria* spp., preventing the development of strong, IFN-γ-driven responses, which have been shown to be crucial for control of *Eimeria* infections (Rothwell et al., 2004). IL-10 was undetectable in the serum of healthy uninfected birds. As expected from previous detection of the IL-10 mRNA in infected birds (Rothwell et al., 2004), the level of circulating IL-10 was substantially increased around 5 days following either low or high dose challenge (Fig. 6). These observations indicate that circulating IL-10 could provide a marker for infection, and that *Eimeria* infections could produce systemic immunosuppression.

### 3.5 The impact of IL-10 neutralisation of T cell and macrophage activation

**Fig. 7:**
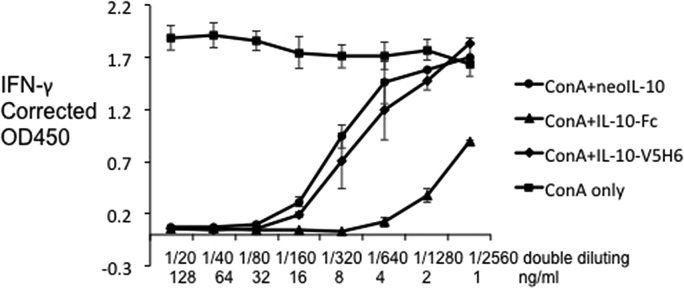
Effect of rchIL-10 on the ability of splenocytes to express IFN-γ after mitogen stimulation. Splenocytes were unstimulated or ConA stimulated with different concentrations of rchIL-10 and controls. All the control proteins showed no effect on ConA stimulation, only results from Ig-tagged control protein was shown in the graph. IFN-γ expression at 72 h was measured by capture ELISA. The doubling dilutions started from a 1/20 dilution for neoIL-10 and 128 ng/ml for rchIL-10-Fc or chIL-10-V5H6.

**Fig. 8:**
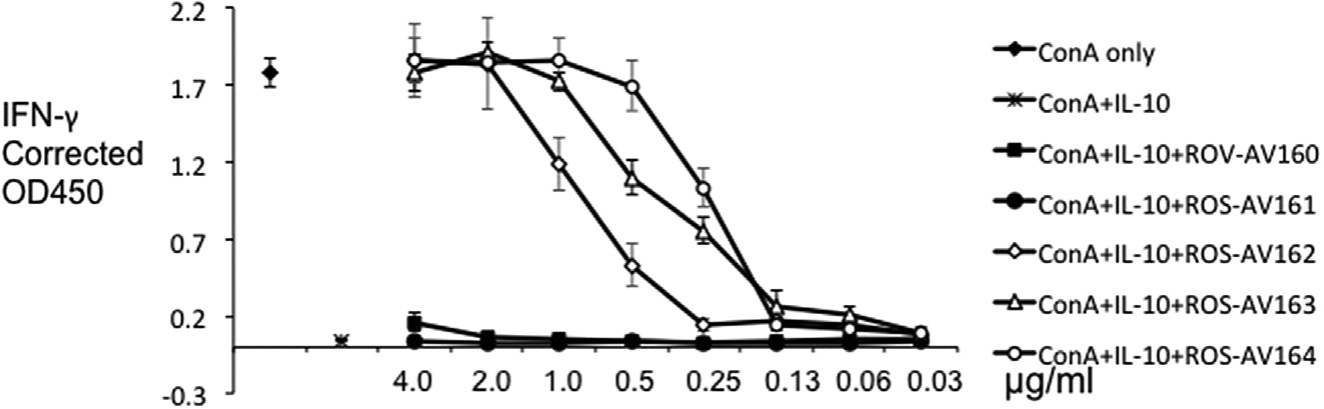
Neutralising effect of mAbs to chIL-10. ChIL-10 was incubated with mAbs for 2 h before IL-10 bioassay as in Fig. 7. Assays were carried out in triplicate, and representative data from three independent experiments is shown.

Mammalian IL-10 strongly inhibited proliferation of CD4^+^ T cells and production of cytokines including IFN-γ, IL-2, IL-4 and TNF-α (Couper et al., 2008, Joss et al., 2000, Moore et al., 2001). As in mammals, chIL-10 inhibits the expression of IFN-γ by mitogen-activated splenocytes (Rothwell et al., 2004). Each of the forms of IL-10 produced for this study inhibited IFN-γ expression by splenocytes after mitogen stimulation (ConA). A 1/80 dilution of neoIL-10 or 32 ng/ml of chIL-10-V5H6 or 8 ng/ml of chIL10-Fc completely inhibited IFN-γ expression (Fig. 7). Preincubation with ROS-AV160 and 161 did not neutralise rchIL-10 even at a concentration of 4 μg/ml. However ROS-AV162, 163 and 164 completely neutralised the inhibition effect by rchIL10 at high concentrations, and this effect titrated out with increasing dilution of mAbs (Fig. 8). These neutralising antibodies provide useful tools to study the regulatory role of IL-10 in chicken immune system as well as the molecular interaction with its receptor.

**Fig. 9:**
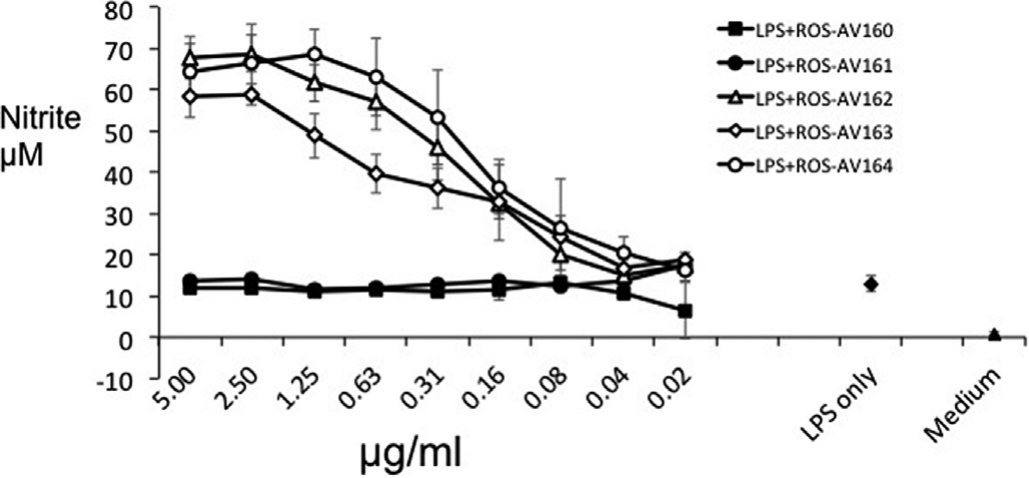
Up-regulation of NO production by mAbs to IL-10. ChBMMs produced NO in response to LPS. Neutralising mAbs to chIL-10 (ROS-AV162, 163 and 164) up-regulated the production of NO by LPS-stimulated chBMMs. Assays were carried out in triplicate, and representative data from three independent experiments is shown.

To test the potential paracrine role of IL-10, implied by the expression in subsets of macrophages above, we tested the impact of adding the neutralising antibodies to stimulated macrophages. Nitric oxide production in mice contributes to macrophage cytotoxicity towards pathogens including parasites, fungi and bacteria. Mouse macrophages produce NO after activation with LPS. This activity is not shared by the macrophages of large animals, including humans and pigs (Kapetanovic et al., 2012, Schroder et al., 2012). IL-10 inhibits NO production of activated mouse macrophages and compromises cytotoxic functions (Gazzinelli et al., 1992, Oswald et al., 1992, Romani et al., 1994). Neutralisation of endogenous IL-10 by anti-IL-10 antibodies up-regulated nitric oxide production in murine macrophages *in vitro* and protected susceptible mice from challenge with *Candida albicans* (Romani et al., 1994). Anti-IL-10 treatment *in vivo* results in a substantial and significant increase in LPS-induced serum NO levels in a mouse model (ter Steege et al., 1998). Like mouse macrophages, chicken macrophages produced abundant NO following activation with LPS or cytokines (Lillehoj and Li, 2004, Sung et al., 1991, Weining et al., 1996). As shown in Fig. 9, this activity is conveniently assayed in chBMMs. Incubation with the neutralising mAbs ROS-AV162, 163 and 164 significantly up-regulated the NO concentrations by LPS-stimulated BMMs in a dose-dependent manner, where the non-neutralising antibodies had no effect.

## 4 Conclusion

We have produced five mouse mAbs to chicken IL-10 using chIL-10-Fc as immunogen. All five monoclonal antibodies recognised specifically chIL-10 via ELISA and WB. Two capture ELISA assays were developed to quantify native IL-10. Three mAbs (ROS-AV162, 163 and 164) neutralised the bioactivities of chIL-10 on ConA stimulated splenocytes and upregulated the nitric oxide production by LPS-stimulated chBMMs. These antibodies are new tools to investigate the important role of IL-10 in the chicken immunity and diseases. The data indicate that IL-10 is immunosuppressive in chickens as it is in mammals, and suggest that intervention in IL-10 production could be used to manipulate chicken immune responses.
